# Factors Affecting Surgical Decision-making—A Qualitative Study

**DOI:** 10.5041/RMMJ.10324

**Published:** 2018-01-29

**Authors:** Caroline Gunaratnam, Mark Bernstein

**Affiliations:** Division of Neurosurgery, Toronto Western Hospital, University Health Network, University of Toronto, Toronto, Canada

**Keywords:** Patient safety, surgery, training

## Abstract

**Background:**

Guidelines and Class 1 evidence are strong factors that help guide surgeons’ decision-making, but dilemmas exist in selecting the best surgical option, usually without the benefit of guidelines or Class 1 evidence. A few studies have discussed the variability of surgical treatment options that are currently available, but no study has examined surgeons’ views on the influential factors that encourage them to choose one surgical treatment over another. This study examines the influential factors and the thought process that encourage surgeons to make these decisions in such circumstances.

**Methods:**

Semi-structured face-to-face interviews were conducted with 32 senior consultant surgeons, surgical fellows, and senior surgical residents at the University of Toronto teaching hospitals. An e-mail was sent out for volunteers, and interviews were audio-recorded, transcribed verbatim, and subjected to thematic analysis using open and axial coding.

**Results:**

Broadly speaking there are five groups of factors affecting surgeons’ decision-making: medical condition, information, institutional, patient, and surgeon factors. When information factors such as guidelines and Class 1 evidence are lacking, the other four groups of factors—medical condition, institutional, patient, and surgeon factors (the last-mentioned likely being the most powerful)—play a significant role in guiding surgical decision-making.

**Conclusions:**

This study is the first qualitative study on surgeons’ perspectives on the influential factors that help them choose one surgical treatment option over another for their patients.

## BACKGROUND

Guidelines and Class 1 evidence are strong factors that surgeons use to help make decisions. With little Class 1 evidence to guide most decisions, dilemmas arise and surgeons often turn to other factors to help guide them make these decisions.^1^,^2^ Experience, training, expertise, research involvement, the availability of equipment and tools, financial constraints, and reimbursement issues are some of the important factors surgeons consider.^1^,^3^–^6^

With the presence of excellent working environments, availability of equipment and surgical tools, and the continuous appearance of new surgical techniques, procedures, and research, the question of which surgery would best treat a specific operable condition is a major challenge for surgeons. This dilemma often invites other important factors to help determine individualized surgical treatment options for each patient.

Few studies have explored the variability of surgical treatment options and compared which procedure may or may not be superior to the others.^7^–^16^ We sought to acquire a better understanding of the factors that influence surgeons’ decision-making when there is a lack of guidelines or Class 1 evidence to help them, using qualitative research methodology.^1^,^3^

## METHODS

The study was approved by the Research Ethics Board at the University Health Network. Informed consent was obtained from all participants.

### Study Design

A prospective qualitative study was conducted to examine surgeons’ views on the influential factors that encourage them to choose one equally fit surgical procedure over another. Participants for this study were consultant surgeons, surgical fellows, and senior surgical residents from various fields within surgery working at academic hospitals of the University of Toronto, Canada. A total of 733 consultant surgeons and clinical fellows in the Department of Surgery at the University of Toronto were invited to participate in this study by email using *E clips*—priority news from the department of surgery at the University of Toronto; two senior surgical residents were specifically requested, as their perspectives would be expected to be valuable, and it added to the spectrum of senior and junior surgeons. Those who were interested to participate e-mailed the co-investigator (C.G.) who scheduled appointments with everyone who responded. Semi-structured face-to-face interviews were conducted with all of the participants using an open-ended questionnaire with specialty-specific clinical vignettes (Appendix). The clinical vignettes were developed based on the most common surgical conditions that currently do not have one single standard surgical approach, guidelines, or Class 1 evidence to guide surgeons.

### Setting and Participants

Participants were either senior or consultant surgeons, surgical fellows, or senior surgical residents in the Department of Surgery working at academic hospitals of the University of Toronto. All participants invited to participate in this study were above 18 years of age, and spoke and understood English well.

### Sample Size

Thirty interviews were sought, a sample size likely to be sufficient for data saturation. Data saturation is a concept used in qualitative research methodology to describe the point at which successive interviews will not likely yield any new themes beyond those already achieved.^15^,^17^

### Data Collection

A single co-investigator (C.G.) conducted an open-ended, face-to-face interview with each participant using a semi-structured guide (Appendix). Themes were explored as they emerged. All interviews were digitally audio-recorded and transcribed. Demographic data such as age, gender, education, and employment were collected. One out of the three clinical vignettes from three different specialties—general surgery, orthopedic surgery, and neurosurgery—was provided to each participant based on their preference and specialty. Responses from the clinical vignettes were collected and examined to determine the presence or absence of diversity in decision-making.

### Data Analysis

Interview responses were collected in tabular form and examined through modified thematic analysis using open and axial coding. Open coding is the deconstruction of information into common groups based on shared ideas, and axial coding involves organizing information into overarching themes.^15^,^17^

### Research Ethics

Participation in this study was entirely voluntary, and informed consent was obtained from all participants. All audiotapes and anonymized transcripts were encrypted and stored securely. This study was approved by the Research Ethics Board at the University Health Network (Toronto).

## RESULTS

A total of 733 emails were delivered; e-mails were sent to 516 consultants (includes scientists and adjunct faculty) of whom 186 opened the e-mail, and to 217 fellows, of whom 131 opened the e-mail. Two senior surgical residents were specifically requested to participate. In total, 32 interviews were conducted. Table 1 shows the details of the demographic data for 32 study participants. Table 2 shows surgeons’ responses to survey questions including the number of participants who felt that their patients’ age influences their surgical decision-making, the number of participants who felt that their experience working as a surgeon has an influence on their surgical decision-making, the number of participants who felt that their personal views in surgical decision-making outweigh the current prevailing methods of treatment done by the majority of other surgeons, the number of participants who felt that their geographical location (i.e. country, city) influences their surgical decision-making, and participants’ views on whether non-financial incentives have an effect on their surgical decision-making.

**Table 1:** Surgeon Demographics.

Category		*n*
Gender	Male	24
	Female	8

Permanent Country of Residence	Canada	25
	International	7

Country of Medical School	Canada	19
	International *	13

Residency Program	General Surgery	7
	Neurosurgery	9
	Orthopedic Surgery	7
	Pediatric General Surgery	1
	Pediatric Plastic and Reconstructive Surgery	4
	Pediatric Urology	4

Years of Surgical Practice	0 †	8
	1–10	12
	11–20	4
	over 21	8

* Surgeons from outside of Canada enrolled in a surgical or research fellowship program at the University of Toronto.

† Six surgical fellows and two senior surgical residents.

**Table 2:** Surgeon’s Responses to Survey Questions.

Question	Yes	No
Does the patient’s age influence surgical decision-making?	28	4
Does surgical experience influence surgical decision-making?	30	2
Does the surgeon’s personal views regarding surgical decision-making outweigh the current prevailing methods of treatment by other surgeons?	13	19
Does the geographical location (country, province, city) influence surgical decision-making?	23	9
Do non-financial incentives play a role in surgical decision-making?	3	29

### Thematic Analysis

Seven over-arching themes were drawn from the interview data and are described below.

#### 1. Patient factors, especially age, are important factors in decision-making

Twenty-eight out of 32 surgeons interviewed felt that patients’ age was an important factor to consider when deciding on a specific treatment option. With all of the advances in technology and anesthesia, many surgical procedures are routinely performed on elderly patients. However, surgeons felt that elderly patients were often given less aggressive or more cautious surgical approaches focused on quality of life as opposed to cure. Alternately, younger patients were often recommended more aggressive surgical approaches focused on cure or longevity.

In pediatric surgery, certain surgical procedures performed at a later stage in the condition, when the patient was older and had matured, produced better and longer-lasting surgical outcomes. Gender was an important factor to consider when the surgical procedure would affect fertility, especially in females, as well as slight variations in surgical approach. For example, women undergoing craniotomy often requested minimal hair shaving, whereas men did not. In orthopedic surgery, women were often found seeking treatment for their knee arthritis at a later stage in the disease, whereas men were found seeking medical attention at an earlier stage and received better outcomes. This may also be linked to cultural differences, where women are often the primary caretakers at home and often give priority to other family members’ health needs over their own. However, surgeons felt that cultural differences did not affect their decision-making but felt that it was important to recognize and respect them. Jehovah’s Witness patients’ non-acceptance of blood transfusion is well established and respected. Aside from that, surgeons felt patients’ religious beliefs did not affect their decision-making, but recognized the importance to respect them. Surgeons also felt that differences in patient personalities did not impact their decision-making but rather their approach to communicating with the patient. Patient preferences were important factors (such as an aversion to surgery), but depended heavily on the clinical situation.

#### 2. Surgeons’ personal factors influence their decision-making

Male and female surgeons did not think that their gender influenced their decision-making. However, female surgeons recognized that they were more sensitive around surgical procedures that directly impacted their patients’ fertility and felt that they were able to understand and relate to their patients better in relation to fertility surgery compared to non-fertility surgery. Cultural differences amongst surgeons did not have any impact on their surgical decision-making. Surgeons who had a strong religious belief felt that it was wrong to allow patients to die in an emergency situation even if the outcomes would not likely provide a favorable quality of life. However, some of these surgeons acknowledged that through experience and wisdom passed down from their mentors, allowing patients to die in specific circumstances would be the best thing to do rather than saving their life and leaving them in a severely compromised state. They also felt that these situations required one to really know who their patients are and what they would have wanted.

Thirty out of 32 surgeons interviewed agreed that their years of experience as a surgeon influenced their surgical decision-making. Less experienced surgeons felt uncomfortable to perform unfamiliar and more complex cases; however, they were very open to learning how to approach and perform specific surgical cases and engage in learning if it could benefit their surgical practice. Junior surgeons also felt more pressure to be up to date with all of the medical literature, advances in technology, and new techniques. Although the majority of junior surgeons preferred the tried and true surgical procedures, there were a few surgeons who felt comfortable and excited to be an early adopter of innovative procedures and would seek help and guidance from senior surgeons when necessary. Senior surgeons felt more familiar and comfortable with performing complex cases as well as seeking help or referring patients to specialized surgeons or colleagues when necessary. As surgeons got more experienced, they were more in favor of becoming sub-specialized in their field of surgery. Some surgeons acknowledged that their own personal views about a disease and its treatment might trump the current prevailing methods of treatment used by the majority of surgeons.

#### 3. Training location influences decision-making

Surgeons thought that where they trained has a great impact on the type of surgeons they become, learning from both positive and negative role models. They also thought that working at an academic hospital impacts their decision-making because the majority of their decisions are based on evidence-based medicine and the availability of guidelines. These surgeons felt that working at an academic hospital provides them with easy access to other surgeons who are sub-specialized experts. Having the support of colleagues, mentors, and even surgical trainees is an important factor that helps surgeons feel more comfortable and prepared to take on challenging surgical cases. With surgical trainees such as residents and fellows, the learning experience for both consultants and trainees is never-ending. In these environments, it is crucial to have weekly morbidity and mortality rounds, where surgeons learn from each other’s complications. All surgeons recognized this as a strong factor that led to better decision-making

#### 4. The diagnosis heavily influences decision-making

All the surgeons thought that the diagnosis, degree of severity or stage of the disease, and medical co-morbidities heavily influence their decision-making. Surgeons often refer their patients to medical consultants and anesthetists to assess fitness for surgery and to optimize their patients’ medical status. Medical co-morbidities are often a factor in all disciplines within surgery that may alter the risk–benefit ratio for a specific surgical patient.

#### 5. Geography, socioeconomics, and resource availability influence decision-making

Surgeons thought that their geographical location influences their decision-making. The country/province/city/hospital the surgeon practices in has influence on the access to resources, such as specific surgical instruments. Surgeons also thought that working at an academic hospital provides them with greater access to such tools and equipment that may not be available in other hospitals. Occasionally these surgeons would receive surgical referrals from non-academic hospitals because of the lack of surgical resources available there and were happy to take on such cases.

#### 6. Surgeons’ comfort or championing of a procedure affect decision-making

Surgeons felt that if a surgical procedure they were very comfortable with produced as good outcomes as other procedures, they would usually select that procedure. Most surgeons were more comfortable with “tried and true” methods, and few surgeons were comfortable being early adopters of novel techniques unless they were the innovator. All surgeons felt very comfortable to offer a procedure they “champion” to their patients, and appreciated the need to be aware of not disadvantaging their patients by doing this. Surgeons also thought that they would be more likely to receive referrals from other doctors whose patients were in need of their specialized care or surgical procedure. If there was a known expert who had significantly better outcomes because he/she used a different technical surgical approach, surgeons said they would not have a problem referring their patients to that expert. Surgeons’ egos do play a role, and many surgeons admitted that their view of a specific condition and how it should be treated could overrule a body of evidence that stated otherwise, except for good-quality Class 3 evidence.

#### 7. Personal gains to the surgeon are not strong factors in decision-making

All surgeons were aware of the potential conflicts of interest in everyday practice, for example, when a surgeon is involved in a clinical trial and it would help the trial reach fruition by recommending a certain procedure. Twenty-nine out of 32 participants felt that receiving a higher reimbursement to perform one surgical procedure over another would not cloud their judgment. However, surgeons were aware that this may be a factor that possibly affects other surgeons, and some were aware of specific examples.

### Responses to the Clinical Vignettes

One clinical vignette in the field of neurosurgery, orthopedic surgery, or general surgery was posed to assess variability in approaches (Appendix). Out of nine neurosurgeons who answered the neurosurgical vignette, there were six different ways recommended to approach the case. Orthopedic surgery had six different ways to approach the same vignette from six different surgeons. Although there were only three general surgeons who answered the general surgery vignette, eight other surgeons who had knowledge and background in general surgery also answered; there were seven unique ways from the 11 surgeons. The clinical vignettes were designed for the sole purpose of capturing diversity among the responses by surgeons and to enhance the concept that decision-making among surgeons is quite variable.

## DISCUSSION

One would think in our modern era of high-tech surgery, where almost anything is possible, that the surgical solution to most problems, common or rare, would be clear, but we are far from this situation. One might surmise that patients would be perplexed and possibly disturbed to know that so many different approaches to their problem exist, rather than one obvious approach agreed on by most surgeons.

The responses to the clinical vignettes are a simple demonstration of how different surgeons make different clinical decisions and how variability in the responses per vignette proves that there is a need within the surgical community for guidance on how to approach decision-making in a more unified and systematic manner.

Medical condition factors consist of diagnosis, prognosis, signs and symptoms, the acuity, and whether the medical condition is a benign or a malignant one. Taking all of these components into consideration is important as it helps surgeons determine the urgency of the treatment and the type of treatment plan or surgical procedure required for their patient.

Information factors include the availability of guidelines or Class 1 evidence on a specific treatment or surgical procedure. The information available to surgeons regarding a specific medical condition and the available surgical procedures constitutes components that help surgeons provide a service to their patients in a timely and more accurate manner.^1^,^13^–^15^,^18^ Having information regarding outcomes, variations in surgical approaches and techniques, and the availability of tried and true surgical methods versus new innovative methods helps surgeons predetermine and identify errors that were made in the past by others and discover ways to prevent them from occurring in their hands, as well as knowing to whom and when to reach out for help by collaborating with other surgeons.^19^–^22^

Institutional factors occasionally determine whether a surgical procedure or treatment plan could be provided to their patients or not, based on the availability of specific surgical tools and expertise.^3^,^6^,^23^–^25^ Surgeons working at academic hospitals of the University of Toronto found that occasionally they would receive referrals from non-academic hospitals due to the unavailability of specific surgical equipment and/or expertise. Although surgeons felt that their institution provided many resources regarding surgical equipment, they expressed a concern that there was still room for improvement.

Patient factors including their personal factors such as age, gender and fertility, cultural backgrounds, religious beliefs, and personalities are factors that surgeons must keep in mind when encountering any patient.^1^–^5^,^26^ This helps the surgeon get a better understanding for their patient as well as help to establish a better rapport with them. Age in particular, whether working with the pediatric age group or with adults and the elderly, is a factor that surgeons still consider. Pediatric surgeons found that certain procedures provided better outcomes to their patients if carried out later on in their development.

Other important patient factors include patients’ medical co-morbidities, past surgical experiences, economic factors (travel and accommodation), role in their family (primary caretaker or primary earner), occupation, their desired lifestyle and how treatment may or may not affect it, patients’ expectation of the surgery and outcomes, patient preferences, and patients’ biases on new innovative surgical procedures—thinking that “newer” means “better.”^17^,^27^–^31^ These factors are all very important for a surgeon to keep in mind and to have open and honest conversations with their patients about. This in turn empowers the patient to be able to make a well-informed decision regarding their possible treatment options, possibilities of complications, and their overall outcomes of the treatment plan, as well as allowing patients and their families to better prepare themselves mentally, emotionally, and financially for the upcoming lifestyle changes and challenges they may have to face in the near future. These factors also help surgeons get a better picture as to how their patients will have to prepare for surgery before and afterwards, allowing surgeons to better assist their patients through this difficult and vulnerable experience in their life.

Surgeon factors play a very significant role in surgeons’ decision-making. These factors include the level and amount of training a surgeon may have in a particular procedure, junior or senior surgeon based on the number of years of experience, general versus sub-specialist, the availability of peer support such as mentors or colleagues to guide or support them during new or unfamiliar surgical procedures, a surgeons’ comfort level and familiarity with a specific procedure, the skill set of each individual surgeon and his surgical team following the “in my hands” concept, and the overall surgical outcomes of a procedure by each surgeon.^4^–^6^ The more a surgeon is familiar with and experienced in a specific procedure (i.e. his/her comfort level), the stronger this factor becomes in the decision-making process. Although surgeons in general prefer the tried and true procedures over newer innovative surgical procedures, they recognized the importance of the newer surgical procedures and appreciated that surgery would never advance without them. However, surgeons are cautious about providing these newer surgical procedures to patients and are aware of patients’ own biases about newer surgical procedures being better.

## CONCLUSION

This study reveals five factors—medical condition, information, institutional, patient, and surgeon factors—in surgical decision-making. It also highlights the importance of surgeons re-evaluating and prioritizing four of those factors when there is a lack of information factors available to guide them during the decision-making process.

## STUDY LIMITATIONS

This was a qualitative study using a subset of surgeons in a large department of surgery, in an academic health science center within a socialized health-care system. The results may not be generalizable to other health-care systems/hospitals.

## Supplementary Information

appendix


