# Supplementary material for: Factors Affecting Surgical Decision-making—A Qualitative Study
Source: Rambam Maimonides Med J. 2018 Jan 29;9(1):e0003. doi: 10.5041/RMMJ.10324 (PMC5796734; doi:10.5041/RMMJ.10324)
Supplement: Supplementary file 1 [file rmmj-9-1-e0003-am.pdf]

SUPPLEMENTARY MATERIAL

This appendix has been provided by the authors to give readers additional background reading and information

Supplement to Factors Affecting Surgical Decision-making—A Qualitative Study

Gunaratnam C, Bernstein M. Factors Affecting Surgical Decision-making—A Qualitative Study. Rambam Maimonides Med J 2018;9 (1):e0003. doi:10.5041/RMMJ.10324

INTERVIEW QUESTIONS

1. How do you make decisions about a particular operation?
2. How much do patients' preferences influence your decisions?
3. Does the personality of a patient affect your final decision-making?
4. How does the gender/cultural background/religion of patients affect your decision-making?
5. How does your gender/cultural background/religion affect your decision-making?
6. Does the age of the patient matter when deciding on a specific treatment option?
7. Do your age and experience have any influence on your decision-making?
8. How do medical co-morbidities affect your decision-making? Please explain.
9. How does the presence of a clinical research study for which your patient is a candidate affect your decision-making?
10. Do non-financial incentives play a role in your decision-making, for example, adding another case to a database of a procedure you plan to publish on?
11. When one type of surgical procedure provides a higher reimbursement, would that have any effect on your choice of procedure?
12. How does the innovation or newness of a procedure affect your decision-making?
13. If you are the champion of a procedure, like the only surgeon in your center who is an "expert" on this surgery, does this affect your decision-making? Do you think it would be beneficial to inform patients about the other techniques and their advantages, or do you think the patient would not have enough medical knowledge to make the best-informed decision about which procedure to choose?

14. If there is an expert in the city who is “better” than you at a specific procedure, would you refer the patient?
15. Do you think where you went to medical school/residency/fellowship programs has an influence on guiding you to make decisions?
16. Does your institution as an academic hospital have any influence on the procedures you choose to do?
17. Does your geographic location (country, city) influence your decision-making?
18. How does the availability of surgical tools/instruments required for a procedure affect your decision to do one procedure over another? If a good methodology was not available in your town but available 200 km away, would you refer the patient?
19. Obviously a surgeon’s personal understanding and perspectives on a particular disease and its treatment weighs extremely heavily in decision-making. For you, could your personal views outweigh the current prevailing methods of treatment by the majority of other surgeons?
20. Looking back at your journey through residency/fellowship programs and years of experience as a surgeon, what would be your advice to new trainees and upcoming surgeons on how to make the best decisions when deciding on surgical treatment options for their patients?

VIGNETTES

Neurosurgery

A 77-year-old woman in good health has a 2-week history of mild right hemiparesis, confusion, and headache. CT shows a 2-cm thick left chronic subdural hematoma with shift.

- Would you do 1 bur hole, 1 mini-craniotomy, or 2 bur holes?
- Would you do the procedure under local anesthesia or general anesthesia?
- Would you leave a drain in or not use a drain?

Orthopedic surgery

A 69-year-old man with severe osteoarthritis of the right hip requires a hip replacement.

- What hardware (i.e. which company’s product) would you use?
- Would you do a hemiarthroplasty or a total hip replacement?
- What bearing surfaces would you use—metal, plastic, or ceramic?
- Would you use cement or not use cement?
- Would you do the procedure under spinal anesthesia or general anesthesia?

General Surgery

A 47-year-old man has a very symptomatic inguinal hernia requiring surgery.

- Would you do it open or endoscopically?
- Would you use mesh or not use mesh?
- Would you do this procedure under general anesthesia or local anesthesia?
